# Nasopharyngeal Carcinoma Progression: Accumulating Genomic Instability and Persistent Epstein–Barr Virus Infection

**DOI:** 10.3390/curroncol29090475

**Published:** 2022-08-23

**Authors:** Xue Liu, Yayan Deng, Yujuan Huang, Jiaxiang Ye, Sifang Xie, Qian He, Yong Chen, Yan Lin, Rong Liang, Jiazhang Wei, Yongqiang Li, Jinyan Zhang

**Affiliations:** 1 Department of Medical Oncology, Guangxi Medical University Cancer Hospital, 71 Hedi Road, Nanning 530021, China; 2 Department of Otolaryngology & Head and Neck, The People’s Hospital of Guangxi Zhuang Autonomous Region, Guangxi Academy of Medical Sciences, 6 Taoyuan Road, Nanning 530021, China; 3 Institute of Biopharmaceutical and Health Engineering, Tsinghua Shenzhen International Graduate School, Tsinghua University, Shenzhen 518055, China

**Keywords:** Epstein–Barr virus, nasopharyngeal carcinoma, genomic instability, mutation

## Abstract

Genomic instability facilitates the evolution of cells, tissues, organs, and species. The progression of human malignancies can be regarded as the accumulation of genomic instability, which confers a high evolutionary potential for tumor cells to adapt to continuous changes in the tumor microenvironment. Nasopharyngeal carcinoma (NPC) is a head-and-neck squamous-cell carcinoma closely associated with Epstein–Barr virus (EBV) infection. NPC progression is driven by a combination of accumulated genomic instability and persistent EBV infection. Here, we present a review of the key characteristics of genomic instability in NPC and the profound implications of EBV infection. We further discuss the significance of profiling genomic instability for the assessment of disease progression and treatment efficacy, as well as the opportunities and challenges of targeted therapies for NPC based on its unique genomic instability.

## 1. Introduction

Nasopharyngeal carcinoma (NPC) is a head-and-neck squamous-cell carcinoma (HNSCC) that originates in the nasopharyngeal epithelium [1]. According to the World Health Organization (WHO) histopathological classification, NPC is classified as keratinizing squamous-cell carcinoma, non-keratinizing carcinoma, and basaloid squamous-cell carcinoma [2]. In addition, non-keratinizing nasopharyngeal carcinoma is subdivided into differentiated non-keratinizing carcinoma (WHO classification, type II) and undifferentiated non-keratinizing carcinoma (WHO classification, type III) [2,3]. NPC has a high incidence in southern China and some Southeast Asian regions, yet is extremely rare in most parts of the world [1]. In areas with a high incidence of NPC in China, more than 90% of patients are histopathologically diagnosed with undifferentiated non-keratinizing squamous-cell carcinoma, and latent Epstein–Barr virus (EBV) infection can be detected in almost all of these cases [1].

The progressive accumulation of genomic instability and persistent EBV infection have encouraged the development of NPC [4]. First, exposure to carcinogens such as nitrosamines and polycyclic aromatic hydrocarbons impairs DNA damage repair and induces a series of molecular genetic pathological alterations in the nasopharyngeal epithelium, which in turn induces genomic instability and creates favorable conditions for EBV infection [5]. The EBV genome, EBV-encoded proteins, and EBV non-coding RNAs further exacerbate genomic instability in the host cells [5]. NPC cells continuously accumulate mutations, maintain EBV infection, enhance their resilience in the tumor microenvironment (TME), and acquire the capacity for malignant biological behaviors such as maintaining dysregulated proliferation, inducing tumor-associated angiogenesis, generating regional invasion, and eventually forming remote metastasis. Exploring the mechanism through which EBV infection promotes genomic instability in host cells could not only provide a comprehensive understanding of NPC pathogenesis, but also provide clinical translational insight for exploring tumor-targeted therapeutic strategies. In this review, we summarize the linkage mechanism between host genomic instability and EBV infection as well as how this cooperation drives the multistep progression of NPC carcinogenesis.

## 2. Characteristics of Genomic Instability in NPC

**Figure 1:**
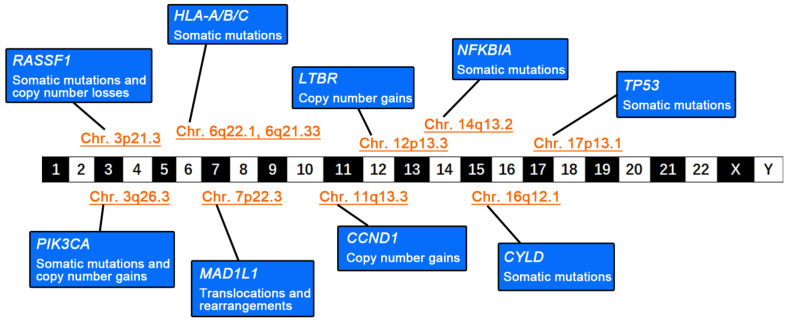
Genomic instability in nasopharyngeal carcinoma (NPC). The schematic diagram summarizes the genetic abnormalities and alterations in various tumor-associated functional genes and their locations on the chromosome.

The characteristics of genomic instability in NPC are well-recognized. Although a wide variety of somatic mutations are found, copy-number variations (CNVs) are more frequent in NPC than in other HNSCCs, as determined by whole-genome sequencing (WGS) [6,7]. During NPC development, functional genes enriched in various signaling pathways are altered through different mutational forms or structural changes (Figure 1), leading to different phenotypic switches in cellular biological functions. During NPC progression, genomic instability is aggravated by somatic mutations, CNVs, and structural changes that gradually accumulate in nasopharyngeal epithelial cells, accompanied by the enhancement of adaptive plasticity in response to the TME. Consequently, cellular malignant transformation becomes irreversible and nasopharyngeal epithelial cells evolve into invasive NPC cells.

### 2.1. Diverse Somatic Mutations

Somatic mutations in oncogene-encoding genes, such as epidermal growth factor receptor *(EGFR*), phosphatidylinositol-4,5-bisphosphate 3-kinase catalytic subunit alpha (*PIK3CA*), Kirsten rat sarcoma viral oncogene homolog (*KRAS*), and Akt serine/threonine kinase 1 (*AKT1*), are more diverse in NPC than in other cancers [7]. Somatic mutations in NPC include missense mutations, nonsense mutations, frameshift mutations, and splicing regions [7]. Functional genes responsible for maintaining genomic stability are frequently mutated in NPC. The tumor protein p53 (*TP53*) gene functions to repair DNA damage and removes cells with unfixable injured genetic integrity by arresting them in the G1/S cell phase [8]. Bruce et al. reported a high rate (28.6%, 20/70) of somatic mutations in *TP53* in patients with NPC [9]. The enrichment of somatic mutations in the classical NF-κB signaling axis is considered the key driver for the malignant transformation of epithelial cells. Dysfunction of the negative regulation of the NF-κB pathway caused by somatic mutations, combined with overactivated NF-κB signaling stimulated by the expression of EBV-encoded latent membrane protein 1 (LMP1), contributes to NPC pathogenesis [10]. Li et al. identified multiple somatic mutations in the genes encoding negative regulatory molecules of the NF-κB pathway in 41% (43/105) of patients with NPC by performing whole-exon sequencing (WES), including the cylindromatosis (*CYLD*), TNF receptor-associated factor 3 (*TRAF3*), and NFKB inhibitor alpha (*NFKBIA*) genes [11]. *CYLD* encodes deubiquitinating enzymes (DUBs), which regulate cell growth and invasiveness by untying K63-linked ubiquitin chains through deubiquitination [12]. *TRAF3* exerts a variety of biological functions by interacting with membrane receptors, E3 ubiquitin ligases, and other molecules [13]. *NFKBIA* negatively regulates NF-κB activity by inhibiting its translocation from the cytoplasm to the nucleus [11]. High-frequency mutations in *CYLD* (10.5%, 11/105), *TRAF3* (8.6%, 9/105), and *NFKBIA* (6.7%, 7/105) lead to constitutive activation of the nonclassical NF-κB pathway in NPC [11]. Frequent somatic mutations in genes involved in the PI3K/Akt/mTOR and MARK signaling pathways, such as phosphatase and tensin homolog (*PTEN*), *PIK3CA*, and fibroblast growth factor receptor 3 (*FGFR3*) have been found in NPC, and the most frequently mutated pathways are considered to be responsible for NPC progression [6,14]. *PIK3CA* encodes the p110α catalytic subunit, which activates Akt signaling and downstream molecules by generating phosphatidylinositol (3,4,5)-triphosphate (PIP3) [15,16]. The majority of mutations in *PIK3CA* (83.3%, 5/6) occur in the E542K, E545K, and H1047 hotspots [17]. It is speculated that the E545K and E542K mutations disrupt the interaction between *PIK3CA* and the p85 regulatory subunit, whereas the H1047 mutation increases the binding affinity of *PIK3CA* for the phosphatidylinositol substrate [17,18,19]. Mutations in *PIK3CA* promote cell proliferation and migration, and inhibit apoptosis in NPC cells [15]. In addition, high-frequency somatic mutations in chromatin-remodeling-related genes (for example, lysine (K)-specific methyltransferase 2C (*KMT2C*)*,* lysine (K)-specific methyltransferase 2D (*KMT2D*), E1A binding protein p300 (*EP300*), and lysine (K)-specific demethylase 5A (*KDM5A*)), have also been documented in NPC [11]. Interestingly, mutations in the EBV genome have also been shown to affect the biological behavior of EBV-infected cells [20]. The expression of mutant 2117-LMP1 results in enhanced cell proliferation and migration ability and weaker cell–cell adhesion in the normal nasopharyngeal epithelial cell line NP69 through the activation of the NF-κB signaling pathway [20]. Other LMP1 variants (Alaskan, China 1, China 2, Med+, Med−, and North Carolina) have also been demonstrated to block cellular contact inhibition in Rat-1 fibroblasts and enhance the motility of human foreskin keratinocytes (HFKs) by stimulating the PI3K-Akt and NF-κB pathways [21]. The conserved mutation of the EBV gene BamH1-A fragment rightward reading frame 1 (*BARF1*) is common in Indonesia; however, its functional role remains poorly understood [22].

### 2.2. High-Frequency CNVs

With the development of high-throughput sequencing, gene chips, and comparative genomic hybridization (CGH) technologies, there is increasing evidence that CNVs constitute the most common genetic variant in NPC. Increased copy numbers of chromosomes 1q, 8p, 8q, 11q, 12p, 12q, 17q, 19p, 19q, 20p, and 20q and copy-number loss of 1p, 3p, 9p, 9q, 11q, 13q, 14q, and 16q are frequently detected in NPC [23,24]. High-frequency copy-number amplification was detected in cyclin D1 (*CCND1*) (73.7%, 28/38) on chromosome 11q and transforming growth factor-beta receptor 2 (*TGFBR2*) (16.7%, 2/12) on chromosome 3p [25,26]. The copy-number loss of cyclin-dependent kinase inhibitor 2A (*CDKN2A*) on chromosome 9p was also frequent (86.8%, 33/38) [25]. The copy-number loss of 3p and 9p is closely associated with latent EBV infection and has been established as a pivotal early molecular event during the evolution of the normal nasopharyngeal epithelium into NPC [27,28]. Genetic damage to tumor suppressors located in these chromosomal regions (such as *CDKN2A*, Ras association domain family member 1 (*RASSF1A*)*,* and TGFBR2) is a key component of genomic instability in NPC [10]. The tumor suppressor gene *RASSF1* regulates mitosis and maintains genomic stability by repressing cell-cycle-related protein cell division cycle 20 (*CDC20*) and microtubule proteins [29,30,31]. *RASSF1A* inhibits self-renewal and tumorigenicity and reduces the invasiveness of NPC cells in vitro [32]. The copy-number loss of *RASSF1A* destabilizes the host genome and is used as a metric to assess the severity of genomic instability in NPC [33]. Alterations in *CCND1* and *CDKN2A* promote NPC progression by disturbing the cell cycle [34,35].

### 2.3. Gene Translocations and Rearrangements

Chromosome breakage and gene rearrangement occur when an organism is exposed to pathological or harmful external environmental factors, resulting in the formation of new gene fragments that lead to the development of malignant neoplastic diseases [36]. Fusion genes with both breakage and rearrangement have been detected in NPC, such as *UBR5*-*ZNF423*, *RARS*-*MAD1L1*, and *FGFR3*-*TACC3* [37,38,39]. The expression of the *UBR5*-*ZNF423* and *RARS*-*MAD1L1* fusion genes enables NPC cells to proliferate and form colonies in vitro and induce tumorigenesis in vivo [37,38]. Despite the low incidence of gene translocations and rearrangements, their functional roles in NPC progression require further clarification.

### 2.4. Unique Genomic Alterations in Relapse Lesions

The genomic instability of recurrent NPC exhibits unique features compared to those of primary tumors. For instance, all mutations of *TRAF3*, *CYLD,* and *NFKBIA* are clonal (100.0%) in recurrent tumors, whereas they only range from 55.6% to 57.9% in primary tumors [11,40]. This suggests that clonal mutations in the NF-κB pathway are closely correlated with the local recurrence of NPC. The *TP53* mutation rate is significantly higher in recurrent samples than in primary tumors (15.2% vs. 6.4%) [11]. Moreover, mutations in genes such as ABL proto-oncogene 1 (*ABL1*), BUB1 mitotic checkpoint serine/threonine kinase B (*BUB1B*), nuclear receptor corepressor 1 (*NCOR1*), chimeric antigen receptors (*CARS*), heat shock protein 90 alpha family class B member 1 (*HSP90AB1*), and nuclear receptor coactivator 1 (*NCOA1*) have been found only in recurrent diseases [11,41]. Mutations in phosphatidylinositol-4,5-bisphosphate 3-kinase catalytic subunit alpha (*PIK3CA*) were only detected in the recurrent lesions (37.5%, 3/8), but none in their paired primary tumors (0%, 0/7) [42]. In addition, gains in cyclin-dependent kinase 2 (*CDK2*) and erb-b2 receptor tyrosine kinase 3 (*ERBB3*) were found only in recurrent lesions (37.5% and 37.5%, respectively) but not in the corresponding primary tumor controls [42]. Somatic mutations enriched in the PI3K and MAPK pathways, such as Harvey-RAS (*HRAS*) and neuroblastoma-RAS (*NRAS*), are considered key factors driving NPC recurrence [11,14]. Tumor suppressor in lung cancer 1 (*TSLC1*) was downregulated or absent in 81% (21/26) of lymph-node metastases, which was significantly higher than that in the primary tumor of NPC (35%, 9/26) [43]. Thymus cell antigen 1 (*THY1*), a tumor suppressor mainly involved in T-cell activation, neurite outgrowth, and cell apoptosis, was downregulated or lost in 74% (20/27) of metastatic lymph nodes, which was significantly higher than that in primary NPC (40%, 17/43) [44,45]. Alterations in fibronectin type III domain containing 3B (*FNDC3B*), cytoplasmic linker-associated protein 1 (*CLASP1*), annexin A1 (*ANXA1*), and C-X-C motif chemokine ligand 10 (*CXCL10*) have been correlated with metastasis in NPC [46]. In addition, mutations in *EP300*, *PIK3CA*, and splicing factor 3b subunit 1 (*SF3B1*) also contribute to metastasis in NPC [7,47]. Specifically, mutations in the BRCA1-associated protein 1 (*BAP1*) are found only in NPC patients with metastatic disease [48].

### 2.5. Deleterious Germline Variants

Although most cases of NPC occur sporadically, family genetic predisposition to NPC is widely recognized. Unlike somatic mutations, germline variants in susceptibility genes contribute to familial nasopharyngeal carcinoma [49]. Deleterious germline variants lead to genomic instability and promote tumor progression by increasing somatic mutations and altering gene expression [49]. Germline variation in NPC mainly focuses on the pathways of DNA damage repair (such as BRCA2 DNA-repair-associated (*BRCA2*), protein kinase, DNA-activated, catalytic subunit (*PRKDC*), mutL homolog 1 (*MLH1*), and lysine methyltransferase 2C (KMT2C)), host defense (macrophage-stimulating 1 receptor *(MST1R*)), EBV virus infection (BCL2-like 12 (*BCL2L12*), NEDD4-like E3 ubiquitin protein ligase (*NEDD4L*)), and NOTCH signaling (notch receptor 1 (*NOTCH1)*, DLL3 delta-like canonical notch ligand 3 (*DLL3*)) [50,51,52,53]. In particular, multiple familial linkage studies have shown that major histocompatibility complex (MHC) class I genes with genetic susceptibility on chromosome 6p are highly associated with NPC, suggesting a critical role for EBV antigen presentation to host immune cells in this disease [33]. Mutations in MHC class I genes, such as NLR family caspase recruitment domain containing 5 (*NLRC5*), human leukocyte antigen A (*HLA-A*), human leukocyte antigen B (*HLA-B*), and human leukocyte antigen C (*HLA-C*), cause disruption of the antigen-presentation machinery and enable NPC cells to escape immune surveillance [33]. Germline variants in *MST1R* have also been identified in NPC, and the *MST1R*-encoded protein is predominantly expressed in tissue-resident macrophages to protect organs from inflammatory injury [50]. However, most germline variations in NPC are focused on a limited number of specific genes, and the lack of large-scale studies limits our understanding of the risk of germline variations in NPC [54].

## 3. Synergy of Host Genomic Instability and EBV Infection

### 3.1. Host Genomic Alterations Enable Stable EBV Infection

EBV infection can be detected in almost all NPC cases with undifferentiated non-keratinizing carcinomas (WHO type III) [1]. However, EBV infection is rarely detected in normal nasopharyngeal epithelial cells [34]. Paradoxically, EBV infection inhibits the proliferation of immortalized nasopharyngeal epithelial cells [34]. The establishment and maintenance of latent EBV infection in nasopharyngeal epithelial cells is a critical step in the malignant transformation of the nasopharyngeal epithelium. Normal nasopharyngeal epithelial cells exposed to carcinogens such as nitrosamines and polycyclic aromatic hydrocarbons induce specific genomic alterations that enable EBV infection [41]. The copy-number loss of *CDKN2A* (86.8%, 33/38) and copy-number increase of *CCND1* (73.7%, 28/38) are crucial genomic alterations in the transformation from nasopharyngeal epithelium to carcinoma [25]. *CDKN2A* inactivates retinoblastoma protein (RB) by binding to cyclin-dependent kinase 4 (CDK4) or cyclin-dependent kinase 6 (CDK6), resulting in cell-cycle arrest [35]. *CCND1* also regulates the cell-cycle transition from the G1 to the S phase by binding to CDK4 or CDK6, which phosphorylates RB and releases E2F transcription factor [55]. The inactivation of *CDKN2A* and the hyperactivation of *CCND1* eliminates the inhibitory effect on cell growth induced by EBV, converts the lytic infection state to a latent infection state, and induces the sustained expression of the latent EBV genome [34,56]. Clonal expansion can be initiated even if stable and persistent latent EBV infection is present only in a single nasopharyngeal epithelial cell [57]. In contrast, EBV-infected cells lose their potential for malignant transformation once the stable expression of the latent EBV genome is not maintained [15].

### 3.2. EBV Genes and Their Products Promote Host Genomic Instability

Integration of the EBV genome into host cells affects the stability of the host genome through various mechanisms. EBV genes and their products include EBV-encoded RNAs (EBERs), LMP1, latent membrane protein 2 (LMP2), EBV nuclear antigen 1 (EBNA1), BamH1 A rightward transcript miRNAs (BART-miRNAs), BamH1 A rightward transcripts (BARTs), and BARF1. EBV cleavage genes encoding proteins (e.g., BGLF5 and BALF3) promote host genomic instability by inducing the formation of micronuclei (MN) and DNA double-strand breaks and inhibiting the repair of damaged DNA. Micronuclei are derived from acentric chromatids and serve as classic markers of genomic instability [58]. BGLF5 has been proven to be a strong inducer of micronucleus formation and DNA damage [58]. BGLF5 causes DNA damage, inhibits DNA repair, and increases chromosomal aberration (CA), microsatellite instability (MSI), and mutation frequency, thereby contributing to the early stages of the malignant transformation of nasopharyngeal epithelial cells [59]. Another EBV cleavage gene encoding the protein BALF3 induces micronucleus formation and DNA strand breaks, promotes cell migration in vitro, and enhances the tumorigenicity of NPC cells in vivo [60]. However, micronucleus formation is not necessary for EBV cleavage of genes and their products to induce genomic instability. The early cleavage gene *BRLF1* promotes genomic instability in NPC cells by interfering with mitosis to induce chromosomal mis-segregation without causing a significant increase in micronuclei [61]. The EBV cleavage gene encoding the protein BGLF4 induces genomic instability by prolonging the S phase of the cell cycle and causing chromosomal abnormalities [62]. In addition, the EBV product BNRF1 triggers centromeric amplification, thereby causing chromosomal instability [63].

The expression of latent EBV genes exacerbates host genome instability. EBNA1 binds to ubiquitin-specific protease 7 (USP7) to form a p53 binding domain that protects infected cells from apoptosis and inhibits DNA repair [64]. EBNA1 also attenuates the repair of the damage repair via p53 [65]. Promyelocytic leukemia (PML) protein maintains genomic stability by regulating cell survival, DNA damage and repair, and *p53* gene-related apoptosis [66]. EBNA1 induces PML protein degradation by binding to USP7 or casein kinase 2 (CK2), thus contributing to genomic instability [65,67,68]. In addition to affecting the stability of the host genome, EBNA1 also maintains the stability of the EBV genome by regulating the replication and mitotic segregation of EBV episomes [67]. Indeed, EBNA1 is a key factor in sustaining EBV genome activation in all EBV-associated malignancies [67]. EBV-encoded LMP1, which is actively expressed in NPC, interferes with DNA damage repair. LMP1 inhibits the expression of DNA-damage-binding protein 1 (DDB1) through the inactivation of forkhead box O3A (FOXO3a) by activating the NF-κB and PI3K/Akt pathways [69]. Additionally, LMP1 activates telomerase through the expression of c-Myc in nasopharyngeal epithelial cells, induces micronucleus formation, and enhances the sensitivity of host cells to pathological factors that induce DNA damage, thereby promoting the accumulation of genomic instability in NPC cells [70,71]. LMP1 also inhibits p53-mediated DNA repair and transcriptional activity through its C-terminal-activating region 1 (CTAR1) [72]. Moreover, the expression of LMP1 in nasopharyngeal epithelial cells results in the impairment of the G2 checkpoint through the activation of defective checkpoint kinase 1 (Chk1) [73].

The EBV genome has been implicated in the methylation of the host genome and the inactivation of suppressor genes [33]. EBV induces the DNA methylation of the host genome by affecting DNA methyltransferases (DNMTs) [33]. LMP1 activates DNMTs through c-Jun NH2-terminal kinase (JNK) signaling, induces the promoter methylation of calmodulin 1 (*CDH-1*), and downregulates calmodulin E (*E*-cadherin) expression [74,75]. LMP1 also disrupts the microtubule structure and induces chromosomal aberrations in nasopharyngeal epithelial cells by repressing *RASS1A* expression [76]. LMP1 manipulates the infected cell cycle by inactivating the suppressor *CDKN2A* by transferring E2F transcription factor 4 (E2F4)/E2F transcription factor 5 (E2F5) and ETS proto-oncogene 2 (Ets2) to the cytoplasm [77]. In addition to LMP1, LMP2A inactivates *PTEN*, another well-characterized suppressor, by phosphorylating the signal transducer and activator of transcription 3 (STAT3) and inducing DNMT1 transcription [78].

**Figure 2:**
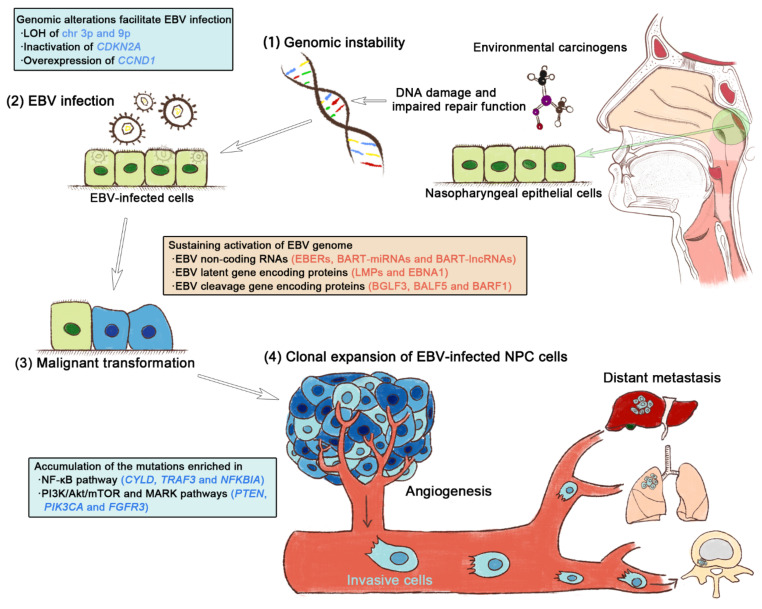
The synergy of host genomic instability and Epstein–Barr virus (EBV) infection drives the multistep NPC progression. (**1**) Regional environmental carcinogens, such as nitrosamines and polycyclic aromatic hydrocarbons, induce DNA damage and repair function impairment, thereby compromising genomic stability. (**2**) Early genetic abnormalities resulting from host genomic instability allow the EBV infection of nasopharyngeal epithelial cells. (**3**) Persistent EBV infection sustainably activates EBV genes and their products and actively contributes to the transformation of non-malignant epithelium to carcinoma. (**4**) The clonal proliferation of EBV-infected cells leads to the accumulation of gene mutations enriched in key oncogenic signaling pathways, eventually triggering the hallmarks of cancer.

In summary, on the one hand, host genomic instability, such as the inactivation of *CDKN2A* and the hyperactivation of *CCND1* provides a prerequisite for latent EBV infection in the nasopharyngeal epithelium. In contrast, the consistent activation of the EBV genome transcript, the expression of EBV-encoded proteins, and the targeting effects of EBV non-coding RNAs on the genome of infected cells further aggravate host genomic instability. The transformation from normal nasopharyngeal epithelium to carcinoma is a continuous, multistep, and complex process. Malignant transformation can be conceptualized as a combined driving model of genomic alteration and EBV infection, in which host genomic instability is at a fundamental and central position (Figure 2). Once EBV latently infects nasopharyngeal epithelial cells, the EBV genes and their related products begin to exhibit the potential to drive malignant cellular transformation [10]. Along with stable EBV infection in the host nasopharyngeal epithelium, EBV-encoded genes and their products deteriorate genomic instability by activating functional gene expression and stimulating the related signaling pathways, which in turn triggers the hallmarks of cancer (Supplementary Table S1) [5]. The oncogenic effects of EBV genes and their products are mediated by the various molecular events of genomic instability that occur during NPC progression, culminating in dysregulated malignant proliferative neoplastic lesions.

## 4. The Clinical Translational Value of Genomic Instability in NPC

Distant metastasis and recurrence are the primary causes of treatment failure and mortality in patients with NPC. Despite significant improvements in the clinical outcomes of NPC patients in recent years, approximately 30% of patients experience tumor relapse or distant metastasis [79]. Research on individualized and precise therapies based on the characteristics of genomic instability is needed to further improve the prognosis of NPC.

### 4.1. Targeted Therapeutic Strategies for Mutated Genes

The therapeutic strategy for *TP53* mutations is to restore the normal physiological function of *TP53* and inhibit the TP53–MDM2 interaction by introducing the wild-type *TP53* gene into tumor cells [8,80,81,82,83]. Gendicine consists of the *TP53* gene and a recombinant adenoviral vector, which is used for the treatment of HNSCCs by ectopically expressing *TP53* to exhibit anti-tumor effects [80]. In addition, the small-molecule compound APR-246 and the novel condensed aminothiourea derivative COTI-2 can reactivate the *TP53* gene by promoting the refolding of the mutated *TP53*, thus restoring the tumor suppressor effect of *TP53* [8]. Mouse double minute 2 (MDM2) promotes tumor progression by inhibiting the transcriptional activity and stability of *TP53* and impairing its ability of *TP53* to induce cell apoptosis [81]. An MDM2 inhibitor, Nutlin-3, is a small-molecule imidazoline analog that exerts antitumor effects by competing with MDM2 for the TP53 binding site and inhibiting p53–MDM2 interactions to reactivate the p53 pathway [83,84]. Nutlin-3 also enhances the sensitivity to cisplatin and upregulates the expression of the apoptosis regulator BCL2-associated X (BAX) and p53-upregulated modulator of apoptosis (PUMA) in C666-1 cells, an NPC cell line [85]. Theoretically, because the majority of mutations in *PIK3CA* are located in the E542K, E545K, and H1047 hot spots, a therapy targeting these mutations can serve as an effective treatment strategy for NPC. However, so far, no selective inhibitors have been developed for the mutations in the hotspots or PI3K inhibitors specific for the p110α subtype [17]. Regarding the most commonly mutated pathway in NPC, a single drug or a combination of drugs targeting the PI3K/Akt/mTOR signaling pathway has been clinically used or studied in preclinical trials. Inhibitors are mainly divided into three categories: PI3K, Akt, and mTOR inhibitors [17]. The PI3K inhibitors alpelisib, copanlisib, duvelisib, idelalisib, and umbralisib have been approved for use in the treatment of breast cancer and hematologic malignancies [86]. However, the clinical benefits of PI3K inhibitors in NPC patients with PIK3CA mutations require further testing [11]. Akt inhibitors (ipatasertib and uprosertib) and mTOR inhibitors (everolimus) have also been investigated in clinical trials in breast cancer [87,88]. The Akt inhibitor MK-2206 was tested in a multicenter phase II study of patients with recurrent or metastatic NPC [89]. In addition, the mTOR inhibitor NVP-BEZ235 and the PI3K-MTOR inhibitor PF-04691502 are currently being tested as new targeted drugs for NPC [90,91]. The *KIT* proto-oncogene), a gene encoding a tyrosine kinase receptor, is frequently mutated or overexpressed in NPC [92,93]. The pharmacological blockage of KIT with imatinib inhibits the proliferation of NPC cell lines in a dose-dependent manner [94]. However, the effect of imatinib in NPC patients with mutated *KIT* remains to be elucidated. Furthermore, *EGFR* and *KRAS* mutations can be used to predict the clinical efficacy of tyrosine kinase inhibitors (TKIs) [95]. However, compared with that in lung cancer, the efficacy of TKIs is limited because of the low mutation rates of *EGFR* and *KRAS* in NPC patients, which is one of the main barriers hampering the development of targeted therapies for the disease [93,96,97].

### 4.2. Genomic Instability and Cancer Immunotherapy

Genomic stability variants such as TMB (tumor mutation burden), MSI, and CNVs are closely associated with the tumor immune microenvironment, and severe genomic instability suggests a poor clinical prognosis in NPC [98]. During the malignant transformation of nasopharyngeal epithelial cells, EBV-infected host cells accumulate somatic mutations, and a small part of the mutated genes are presented to MHC to form tumor neoantigens and are recognized by activated CD^8+^ T cells, stimulating the tumor immune response [99]. TMB is defined as the number of non-synonymous single-nucleotide variants (SNVs) per megabase, which indirectly reflects the ability of tumors to produce neoantigens and can be used to predict the effectiveness of treatment with immune checkpoint inhibitors (ICIs) [99]. ICIs include programmed death-1/programmed death-ligand 1 (PD-1/PD-L1) and cytotoxic T-lymphocyte-associated antigen-4 (CTLA-4) inhibitors, which can restore the immune response against tumor-derived antigens by restoring T-cell activity and promoting the T-cell recognition of tumor-derived antigens [100,101]. Growing evidence indicates that TMB is positively correlated with tumor-derived neoantigen load and high-TMB (TMB-H) cases are more likely to benefit from ICI treatment [101]. A genome-wide analysis of 190 patients with NPC showed that high TMB was strongly associated with EBV infection and that the degree of TMB was significantly correlated with each subtype of NPC [102]. Low TMB limits the production of tumor-derived antigens through somatic mutations, resulting in a lack of tumor neoantigens on the surface of NPC cells and decreased sensitivity to cancer immunotherapy [103]. However, neither tolipalimab monotherapy in the Polaris-02 study nor camlizumab monotherapy in the CAPTAIN study showed a significant correlation between TMB levels and treatment response in NPC [104,105]. Thus, the clinical significance of TMB for predicting prognosis or evaluating treatment efficacy is still controversial, as TMB in NPC is usually lower than that in other cancers [106]. In addition, the value of TMB for assessing the responsiveness of ICIs in NPC requires further validation.

DNA mismatch repair (*MMR*) genes are mainly responsible for correcting DNA replication errors to maintain genomic stability, whereas MMR mutations often lead to MSI [107]. As an important indicator of genomic instability, high microsatellite instability (MSI-H) or MMR functional deficiency (dMMR) is generally accompanied by high TMB [108]. Chen et al. reported that at least one of the four *MMR* gene-encoded mismatch repair proteins, including MLH1, MSH2, MSH6, and PMS2, was lost in 49.3% (34/69) of pre-treatment NPC biopsies [109]. Pembrolizumab, a PD-1 inhibitor used to treat patients with MSI-H/dMMR and unresectable or metastatic solid tumors, has shown proven efficacy in colorectal cancer, melanoma, and NSCLC [110,111,112]. An overall response rate (ORR) of 17.7% (34/192) was observed in a multicenter, non-randomized, phase Ib trial in patients with recurrent/metastatic HNSCC treated with pembrolizumab (KEYNOTE-012) [113]. However, the efficacy of pembrolizumab in the treatment of NPC remains to be evaluated in randomized controlled clinical trials.

Somatic CNVs, also known as segmental aneuploidies, are associated with responsiveness to cancer immunotherapy [114,115]. Patients with relatively frequent genomic amplification of the genes on chromosome 11q13, such as *CCND1*, *FGF14*, *FGF3*, and *FGF4*, as well as ETV6 genomic alterations, exhibit poor clinical response to toripalimab [105,116]. In another clinical trial of patients with metastatic melanoma treated with ipilimumab, a monoclonal antibody against CTLA-4, a negative correlation between CNV frequency and survival rate was established (hazard ratio = 2.24, *p* = 0.0004) [115]. The frequency of CNVs in HNSCC is strongly correlated with T-cell infiltration [117]. Two phase I clinical trials showed a significant benefit of anti-PD-1 therapy for long-term survival in patients with recurrent/metastatic NPC, and copy-number loss in granzyme genes leads to resistance to ICIs and is associated with a reduced survival rate [106].

## 5. Conclusions and Perspective

Genomic instability is an intrinsic driving force that promotes the progression of NPC. An in-depth exploration of the underlying mechanism through which genomic instability occurs, especially in terms of how EBV infection is implicated in host genomic instability, will enable a better understanding of the unique pathogenesis of NPC. The characterization of genomic instability could provide a theoretical basis for the development of novel approaches and strategies for the diagnosis and treatment of NPC. The detection of genomic-instability-related indicators (such as TMB, MSI, and CNVs) allows for a comprehensive evaluation of the effectiveness of immunotherapy for NPC. Targeted therapy based on genomic instability, particularly the genomic instability characteristics of recurrent and metastatic lesions, is crucial for precision NPC therapy in the future. However, the complexity of genomic instability and individual genomic variability are the greatest challenges in precision therapy for NPC.

In the past decade, much work has been carried out to clarify the interaction between the host genome and EBV infection, which consistently contributes to NPC pathogenesis. However, a model of genomic alteration integrated with EBV infection that drives TME evolution allowing NPC progression remains to be established. Solving this mystery could provide valuable biomarkers and effective targets for the development of treatment strategies, especially immunotherapy, for NPC.

## Supplementary Materials

The following supporting information can be downloaded from https://www.mdpi.com/article/10.3390/curroncol29090475/s1. Table S1: Hallmarks of cancer triggered by EBV genes and their products [64,70,74,118,119,120,121,122,123,124,125,126,127,128,129,130,131,132,133,134,135,136,137,138,139,140,141,142,143,144,145,146,147,148,149,150,151,152,153,154,155,156,157,158,159,160,161,162,163].
