# Supplementary material for: Nasopharyngeal Carcinoma Progression: Accumulating Genomic Instability and Persistent Epstein–Barr Virus Infection
Source: Curr Oncol. 2022 Aug 23;29(9):6035–52. doi: 10.3390/curroncol29090475 (PMC9498130; doi:10.3390/curroncol29090475)
Supplement: Supplementary file 1 [file curroncol-29-00475-s001.zip › curroncol-1843632-Supplementary.pdf]

Current Oncology

MDPI

Review

Nasopharyngeal Carcinoma Progression: Accumulating Genomic Instability and Persistent Epstein–Barr Virus Infection

Xue Liu 1, Yayan Deng 1, Yujuan Huang 1, Jiaxiang Ye 1, Sifang Xie 2, Qian He 3, Yong Chen 1, Yan Lin 1 , Rong Liang 1, JiazhangWei 2 , Yongqiang Li 1,* and Jinyan Zhang 1,*

1 Department of Medical Oncology, Guangxi Medical University Cancer Hospital, 71 Hedi Road, Nanning 530021, China

2 Department of Otolaryngology & Head and Neck, The People's Hospital of Guangxi Zhuang Autonomous Region, Guangxi Academy of Medical Sciences, 6 Taoyuan Road, Nanning 530021, China

3 Institute of Biopharmaceutical and Health Engineering, Tsinghua Shenzhen International Graduate School, Tsinghua University, Shenzhen 518055, China

* Correspondence: liyongqiang@gxmu.edu.cn (Y.L.); zhangjinyan@gxmu.edu.cn (J.Z.)

Table S1. The hallmarks of cancer triggered by EBV genes and their products.

EBV genes and products	Targeted molecules	Functions of the targeted molecules	Key regulators/pathways	The hallmarks of cancer	Ref.
LMP1	Id1 and Id3	Regulation of cell cycle	N/A	Sustaining proliferative signaling	[1]
	Cyclin D1	Regulation of cell cycle	EGFR, TIF2, and STAT3	Sustaining proliferative signaling and resisting cell death	[2,3]
	p16	Regulation of cell cycle	N/A	Sustaining proliferative signaling	[4]
	LKB1-AMPK	Regulation of cellular metabolism	MEK/ERK-MAPK signaling pathway	Sustaining proliferative signaling and reprogramming of energy metabolism	[5]
	TGF-β	Regulation of cell proliferation and differentiation	Id1	Evading growth suppressors	[6]
	TP53	Repair of DNA damage and inducing apoptosis	MAP kinases, anti-apoptotic genes *bcl-2* and *A20*	Resisting cell death	[7,8]
	Telomerase	Regulation of replication	c-Myc pathway	Enabling replicative immortality	[9]
	HIF1	Serving as a principal oxygen-sensing transcription factor	prolyl hydroxylase 1 and prolyl hydroxylase 3	Inducing angiogenesis	[10]
	HIF-1/VEGF	Regulation of glucose uptake, metabolism,	JNKs/c-Jun signaling and p42/p44 MAPK pathways	Inducing angiogenesis	[11,12]

	apoptosis, growth, angiogenesis, invasion, and metastasis			
FGF-2	Regulation of angiogenesis and mitosis	NF-κB signaling pathway	Inducing angiogenesis	[13]
VEGF and PGE2	Regulation of angiogenesis	COX-2	Inducing angiogenesis	[14,15]
RAGEs	Regulation of angiogenesis	NF-κB signaling pathway	Inducing angiogenesis	[16]
VEGF	Regulation of apoptosis, angiogenesis, and cell migration	SOCE, JAK/STAT3, and MEK1/ERK1/2 pathways	Activating invasion and metastasis, inducing angiogenesis	[17,18]
Twist	Inducing EMT	NF-κB signaling pathway	Activating invasion and metastasis	[19]
CDH6	Regulation of EMT	miRNA-203	Activating invasion and metastasis	[20]
HIF1α	Regulation of angiogenesis, invasion, and metastasis	Exosome-mediated signaling	Activating invasion and metastasis	[21]
CRT	Maintaining intracellular Ca^{2+} homeostasis	Smad3-dependent TGF-β signaling pathway	Activating invasion and metastasis	[22]
E-cadherin	Maintenance of intercellular adhesion	DNA methyltransferases	Activating invasion and metastasis	[23]
Snail	Regulation of EMT by repressing E-cadherin	IGF1-mTORC2-PDHE1α signaling pathway	Activating invasion and metastasis, reprogramming of energy metabolism	[24,25]
uPA	Degrading extracellular matrix components	Ets1	Activating invasion and metastasis	[26]
MMP-1	Promotion of invasion by breaking ECMs	Ets	Activating invasion and metastasis	[27]
MMP-9	Promotion of invasion by breaking ECMs	c-Jun/Ets1, NF-κB, and AP-1	Activating invasion and metastasis	[15,28-30]
OXPHOS complexes	Oxidative phosphorylation	DNMTs	Reprogramming of energy metabolism	[31]
Glut-1	Regulation of aerobic glycolysis	mTORC1/NF-κB signaling pathway	Reprogramming of energy metabolism	[32]
HK2	Modulation of LMP1-induced glycolysis	c-Myc	Reprogramming of energy metabolism and resisting cell death	[33]

	Th1 lymphocytes	Modulation of immune response	galectin-9/Tim-3	Evading immune destruction	[34]
	VEGF	Regulation of angiogenesis	PI3K/Akt/mTOR/HIF-1α pathway	Inducing angiogenesis	[35]
	ITGα6	Maintenance of epithelial structure	N/A	Activating invasion and metastasis	[36]
	αv-integrin	Regulation of cell migration	ITAM/Syk and Akt signaling pathways	Activating invasion and metastasis	[37]
LMP2A	Transcription factor *p63*	Regulation of epithelial cell differentiation	N/A	Sustaining proliferative signaling	[38]
	S1P	Regulation of proliferation, survival, migration, apoptosis, and chemoresistance	SPHK1	Activating invasion and metastasis	[39]
	TGF-β1	Regulation of cell growth and differentiation	PI3K/Akt pathway	Sustaining proliferative signaling and resisting cell death	[40]
	E-cadherin and N-cadherin	Regulation of EMT	TGF-β1/miR-200/ZEB pathway	Activating invasion and metastasis	[41]
EBNA1	p53-Mdm2	Regulation of apoptosis	USP7/HAUSP	Sustaining proliferative signaling and resisting cell death	[42]
	Survivin	Regulation of apoptosis and cell cycle	Sp1	Resisting cell death	[43]
EBV-miR-BART1	PTEN	Regulation of cell growth and survival	PI3K/Akt, FAK-p130^{Cas}, and Shc-MAPK/ERK1/2 pathways	Activating invasion and metastasis	[44,45]
EBV-miR-BART5	PUMA	Promotion of apoptosis	N/A	Resisting cell death	[46]
EBV-miR-BART6-3p	IFN-β	Modulation of immune response	RIG-I mRNA 22 and RIG-I-like receptor	Evading immune destruction	[44]
EBV-miR-BART7	MICA	Modulation of immune response	TGFβ1/c-Myc	Evading immune destruction	[47]
EBV-miR-BART7-3p	Snail and β-catenin	Regulation of EMT	PI3K/Akt/GSK-3β pathway	Activating invasion and metastasis	[44]
EBV-miR-BART10-3p	β-catenin and Snail	Regulation of EMT	BTRC	Activating invasion and metastasis	[44]
EBV-miR-BART11	NF-κB and TAM	Regulation of proliferation	FOXP1	Staining proliferative signaling	[48]
EBV-miR-BART13-3p	Slug	Regulation of EMT by repressing E-cadherin	c-Jun/Slug signaling pathway	Activating invasion and metastasis	[49]

Abbreviations: Id1, Id proteins 1; Id3, Id proteins 3; EGFR, epidermal growth factor receptor; TIF2, transcriptional intermediary factor 2; STAT3, signal transducer and activator of transcription 3; TGF-β, transforming growth factor beta; HIF1, hypoxia-inducible factor 1; VEGF, vascular endothelial growth factor; FGF-2, fibroblast growth factor 2; PGE2, prostaglandin E2; COX-2,

cyclooxygenase-2; RAGEs, the receptor for advanced glycation end products; SOCE, store-operated Ca(2+) entry; EMT, epithelial-mesenchymal transition; CDH6, cadherin 6; CRT, calreticulin; uPA , urokinase type plasminogen activator; Ets, E26 transformation specific; MMP, matrix metalloproteinase; OXPHOS, oxidative phosphorylation; Glut-1, Glucose Transporter 1; ECM, sextracellular matrix; DNMTs, DNA methyltransferases; HK2, hexokinase 2; ITGα6, integrin-alpha-6; PI3K, Phosphatidylinositol 3-kinase; S1P, sphingosine-1-Phosphate; ZEB1, zinc finger E-box binding homeobox 1; USP7, ubiquitin specific protease 7; HAUSP, herpesvirus-associated ubiquitin-specific protease; Sp1, specificity protein 1; MICA, major histocompatibility complex class I chain-related peptide A; TGFβ1, transforming growth factor-β; BTRC, Beta-transducin repeat containing E3 ubiquitin protein ligase; TAM, tumor-associated macrophages; FOXP1, forkhead box P1; N/A, not available.

References

1. Everly, D.N., Jr.; Mainou, B.A.; Raab-Traub, N. The ID proteins contribute to the growth of rodent fibroblasts during LMP1-mediated transformation. *Virology* **2008**, *376*, 258-269, doi:10.1016/j.virol.2007.12.003.
2. Shi, Y.; Tao, Y.; Jiang, Y.; Xu, Y.; Yan, B.; Chen, X.; Xiao, L.; Cao, Y. Nuclear epidermal growth factor receptor interacts with transcriptional intermediary factor 2 to activate cyclin D1 gene expression triggered by the oncoprotein latent membrane protein 1. *Carcinogenesis* **2012**, *33*, 1468-1478, doi:10.1093/carcin/bgs171.
3. Xu, Y.; Shi, Y.; Yuan, Q.; Liu, X.; Yan, B.; Chen, L.; Tao, Y.; Cao, Y. Epstein-Barr Virus encoded LMP1 regulates cyclin D1 promoter activity by nuclear EGFR and STAT3 in CNE1 cells. *J Exp Clin Cancer Res* **2013**, *32*, 90, doi:10.1186/1756-9966-32-90.
4. Yang, X.; He, Z.; Xin, B.; Cao, L. LMP1 of Epstein-Barr virus suppresses cellular senescence associated with the inhibition of p16INK4a expression. *Oncogene* **2000**, *19*, 2002-2013, doi:10.1038/sj.onc.1203515.
5. Lo, A.K.; Lo, K.W.; Ko, C.W.; Young, L.S.; Dawson, C.W. Inhibition of the LKB1-AMPK pathway by the Epstein-Barr virus-encoded LMP1 promotes proliferation and transformation of human nasopharyngeal epithelial cells. *J Pathol* **2013**, *230*, 336-346, doi:10.1002/path.4201.
6. Lo, A.K.; Dawson, C.W.; Lo, K.W.; Yu, Y.; Young, L.S. Upregulation of Id1 by Epstein-Barr virus-encoded LMP1 confers resistance to TGFbeta-mediated growth inhibition. *Mol Cancer* **2010**, *9*, 155, doi:10.1186/1476-4598-9-155.
7. Li, L.; Guo, L.; Tao, Y.; Zhou, S.; Wang, Z.; Luo, W.; Hu, D.; Li, Z.; Xiao, L.; Tang, M.; et al. Latent membrane protein 1 of Epstein-Barr virus regulates p53 phosphorylation through MAP kinases. *Cancer Lett* **2007**, *255*, 219-231, doi:10.1016/j.canlet.2007.04.014.
8. Fries, K.L.; Miller, W.E.; Raab-Traub, N. Epstein-Barr virus latent membrane protein 1 blocks p53-mediated apoptosis through the induction of the A20 gene. *J Virol* **1996**, *70*, 8653-8659, doi:10.1128/JVI.70.12.8653-8659.1996.
9. Yang, J.; Deng, X.; Deng, L.; Gu, H.; Fan, W.; Cao, Y. Telomerase activation by Epstein-Barr virus latent membrane protein 1 is associated with c-Myc expression in human nasopharyngeal epithelial cells. *J. Exp. Clin. Cancer Res.* **2004**, *23*, 495–506.
10. Kondo, S.; Seo, S.Y.; Yoshizaki, T.; Wakisaka, N.; Furukawa, M.; Joab, I.; Jang, K.L.; Pagano, J.S. EBV latent membrane protein 1 up-regulates hypoxia-inducible factor 1alpha through Siah1-mediated down-regulation of prolyl hydroxylases 1 and 3 in nasopharyngeal epithelial cells. *Cancer Res* **2006**, *66*, 9870-9877, doi:10.1158/0008-5472.CAN-06-1679.
11. Yang, L.; Liu, L.; Xu, Z.; Liao, W.; Feng, D.; Dong, X.; Xu, S.; Xiao, L.; Lu, J.; Luo, X.; et al. EBV-LMP1 targeted DNAzyme enhances radiosensitivity by inhibiting tumor angiogenesis via the JNKs/HIF-1 pathway in nasopharyngeal carcinoma. *Oncotarget* **2015**, *6*, 5804-5817, doi:10.18632/oncotarget.3331.
12. Wakisaka, N.; Kondo, S.; Yoshizaki, T.; Murono, S.; Furukawa, M.; Pagano, J.S. Epstein-Barr virus latent membrane protein 1 induces synthesis of hypoxia-inducible factor 1 alpha. *Mol Cell Biol* **2004**, *24*, 5223-5234, doi:10.1128/MCB.24.12.5223-5234.2004.
13. Wakisaka, N.; Murono, S.; Yoshizaki, T.; Furukawa, M.; Pagano, J.S. Epstein-barr virus latent membrane protein 1 induces and causes release of fibroblast growth factor-2. *Cancer Res* **2002**, *62*, 6337-6344.
14. Murono, S.; Inoue, H.; Tanabe, T.; Joab, I.; Yoshizaki, T.; Furukawa, M.; Pagano, J.S. Induction of cyclooxygenase-2 by Epstein-Barr virus latent membrane protein 1 is involved in vascular endothelial growth factor production in nasopharyngeal carcinoma cells. *Proc Natl Acad Sci U S A* **2001**, *98*, 6905-6910, doi:10.1073/pnas.121016998.
15. Yoshizaki, T. Promotion of metastasis in nasopharyngeal carcinoma by Epstein-Barr virus latent membrane protein-1. *Histol Histopathol* **2002**, *17*, 845-850, doi:10.14670/HH-17.845.
16. Tsuji, A.; Wakisaka, N.; Kondo, S.; Murono, S.; Furukawa, M.; Yoshizaki, T. Induction of receptor for advanced glycation end products by EBV latent membrane protein 1 and its correlation with angiogenesis and cervical lymph node metastasis in nasopharyngeal carcinoma. *Clin Cancer Res* **2008**, *14*, 5368-5375, doi:10.1158/1078-0432.CCR-08-0198.

17. Wei, J.; Zhang, J.; Si, Y.; Kanada, M.; Zhang, Z.; Terakawa, S.; Watanabe, H. Blockage of LMP1-modulated store-operated Ca(2+) entry reduces metastatic potential in nasopharyngeal carcinoma cell. *Cancer Lett* **2015**, *360*, 234-244, doi:10.1016/j.canlet.2015.02.032.
18. Wang, Z.; Luo, F.; Li, L.; Yang, L.; Hu, D.; Ma, X.; Lu, Z.; Sun, L.; Cao, Y. STAT3 activation induced by Epstein-Barr virus latent membrane protein1 causes vascular endothelial growth factor expression and cellular invasiveness via JAK3 And ERK signaling. *Eur J Cancer* **2010**, *46*, 2996-3006, doi:10.1016/j.ejca.2010.07.008.
19. Horikawa, T.; Yang, J.; Kondo, S.; Yoshizaki, T.; Joab, I.; Furukawa, M.; Pagano, J.S. Twist and epithelial-mesenchymal transition are induced by the EBV oncoprotein latent membrane protein 1 and are associated with metastatic nasopharyngeal carcinoma. *Cancer Res* **2007**, *67*, 1970-1978, doi:10.1158/0008-5472.CAN-06-3933.
20. Zuo, L.L.; Zhang, J.; Liu, L.Z.; Zhou, Q.; Du, S.J.; Xin, S.Y.; Ning, Z.P.; Yang, J.; Yu, H.B.; Yue, W.X.; et al. Cadherin 6 is activated by Epstein-Barr virus LMP1 to mediate EMT and metastasis as an interplay node of multiple pathways in nasopharyngeal carcinoma. *Oncogenesis* **2017**, *6*, 402, doi:10.1038/s41389-017-0005-7.
21. Aga, M.; Bentz, G.L.; Raffa, S.; Torrisi, M.R.; Kondo, S.; Wakisaka, N.; Yoshizaki, T.; Pagano, J.S.; Shackelford, J. Exosomal HIF1alpha supports invasive potential of nasopharyngeal carcinoma-associated LMP1-positive exosomes. *Oncogene* **2014**, *33*, 4613-4622, doi:10.1038/onc.2014.66.
22. Ye, D.; Zhu, J.; Zhao, Q.; Ma, W.; Xiao, Y.; Xu, G.; Zhang, Z. LMP1 Up-regulates Calreticulin to Induce Epithelial-mesenchymal Transition via TGF-beta/Smad3/NRP1 Pathway in Nasopharyngeal Carcinoma Cells. *J Cancer* **2020**, *11*, 1257-1269, doi:10.7150/jca.37415.
23. Tsai, C.L.; Li, H.P.; Lu, Y.J.; Hsueh, C.; Liang, Y.; Chen, C.L.; Tsao, S.W.; Tse, K.P.; Yu, J.S.; Chang, Y.S. Activation of DNA methyltransferase 1 by EBV LMP1 Involves c-Jun NH(2)-terminal kinase signaling. *Cancer Res.* **2006**, *66*, 11668–11676. https://doi.org/10.1158/0008-5472.CAN-06-2194.
24. Zhang, J.; Jia, L.; Liu, T.; Yip, Y.L.; Tang, W.C.; Lin, W.; Deng, W.; Lo, K.W.; You, C.; Lung, M.L.; et al. mTORC2-mediated PDHE1alpha nuclear translocation links EBV-LMP1 reprogrammed glucose metabolism to cancer metastasis in nasopharyngeal carcinoma. *Oncogene* **2019**, *38*, 4669-4684, doi:10.1038/s41388-019-0749-y.
25. Horikawa, T.; Yoshizaki, T.; Kondo, S.; Furukawa, M.; Kaizaki, Y.; Pagano, J.S. Epstein-Barr Virus latent membrane protein 1 induces Snail and epithelial-mesenchymal transition in metastatic nasopharyngeal carcinoma. *Br J Cancer* **2011**, *104*, 1160-1167, doi:10.1038/bjc.2011.38.
26. Kim, K.R.; Yoshizaki, T.; Miyamori, H.; Hasegawa, K.; Horikawa, T.; Furukawa, M.; Harada, S.; Seiki, M.; Sato, H. Transformation of Madin-Darby canine kidney (MDCK) epithelial cells by Epstein-Barr virus latent membrane protein 1 (LMP1) induces expression of Ets1 and invasive growth. *Oncogene* **2000**, *19*, 1764-1771, doi:10.1038/sj.onc.1203502.
27. Kondo, S.; Wakisaka, N.; Schell, M.J.; Horikawa, T.; Sheen, T.S.; Sato, H.; Furukawa, M.; Pagano, J.S.; Yoshizaki, T. Epstein-Barr virus latent membrane protein 1 induces the matrix metalloproteinase-1 promoter via an Ets binding site formed by a single nucleotide polymorphism: enhanced susceptibility to nasopharyngeal carcinoma. *Int J Cancer* **2005**, *115*, 368-376, doi:10.1002/ijc.20849.
28. Zeng, L.; Liu, Y.P.; Tao, Y.G.; Ai, M.D.; Zhao, X.R.; Cao, Y. [Cross-talk between c-Jun/Ets1 involved in EB virus-encoded latent membrane protein 1 regulates expression of matrix metalloproteinase-9 in nasopharyngeal carcinoma]. *Zhonghua Zhong Liu Za Zhi* **2005**, *27*, 204-208.
29. Horikawa, T.; Yoshizaki, T.; Sheen, T.S.; Lee, S.Y.; Furukawa, M. Association of latent membrane protein 1 and matrix metalloproteinase 9 with metastasis in nasopharyngeal carcinoma. *Cancer* **2000**, *89*, 715-723, doi:10.1002/1097-0142(20000815)89:4<715::aid-cncr1>3.0.co;2-9.
30. Himelstein, B.P.; Lee, E.J.; Sato, H.; Seiki, M.; Muschel, R.J. Transcriptional activation of the matrix metalloproteinase-9 gene in an H-ras and v-myc transformed rat embryo cell line. *Oncogene* **1997**, *14*, 1995-1998, doi:10.1038/sj.onc.1201012.
31. Luo, X.; Hong, L.; Cheng, C.; Li, N.; Zhao, X.; Shi, F.; Liu, J.; Fan, J.; Zhou, J.; Bode, A.M.; et al. DNMT1 mediates metabolic reprogramming induced by Epstein-Barr virus latent membrane protein 1 and reversed by grifolin in nasopharyngeal carcinoma. *Cell Death Dis* **2018**, *9*, 619, doi:10.1038/s41419-018-0662-2.
32. Zhang, J.; Jia, L.; Lin, W.; Yip, Y.L.; Lo, K.W.; Lau, V.M.Y.; Zhu, D.; Tsang, C.M.; Zhou, Y.; Deng, W.; et al. Epstein-Barr Virus-Encoded Latent Membrane Protein 1 Upregulates Glucose Transporter 1 Transcription via the mTORC1/NF-kappaB Signaling Pathways. *J Virol* **2017**, *91*, doi:10.1128/JVI.02168-16.
33. Xiao, L.; Hu, Z.Y.; Dong, X.; Tan, Z.; Li, W.; Tang, M.; Chen, L.; Yang, L.; Tao, Y.; Jiang, Y.; et al. Targeting Epstein-Barr virus oncoprotein LMP1-mediated glycolysis sensitizes nasopharyngeal carcinoma to radiation therapy. *Oncogene* **2014**, *33*, 4568-4578, doi:10.1038/onc.2014.32.

34. Klibi, J.; Niki, T.; Riedel, A.; Pioche-Durieu, C.; Souquere, S.; Rubinstein, E.; Le Moulec, S.; Guigay, J.; Hirashima, M.; Guemira, F.; et al. Blood diffusion and Th1-suppressive effects of galectin-9-containing exosomes released by Epstein-Barr virus-infected nasopharyngeal carcinoma cells. *Blood* **2009**, *113*, 1957-1966, doi:10.1182/blood-2008-02-142596.
35. Xiang, T.; Lin, Y.X.; Ma, W.; Zhang, H.J.; Chen, K.M.; He, G.P.; Zhang, X.; Xu, M.; Feng, Q.S.; Chen, M.Y.; et al. Vasculogenic mimicry formation in EBV-associated epithelial malignancies. *Nat Commun* **2018**, *9*, 5009, doi:10.1038/s41467-018-07308-5.
36. Pegtel, D.M.; Subramanian, A.; Sheen, T.S.; Tsai, C.H.; Golub, T.R.; Thorley-Lawson, D.A. Epstein-Barr-virus-encoded LMP2A induces primary epithelial cell migration and invasion: possible role in nasopharyngeal carcinoma metastasis. *J Virol* **2005**, *79*, 15430-15442, doi:10.1128/JVI.79.24.15430-15442.2005.
37. Fotheringham, J.A.; Coalson, N.E.; Raab-Traub, N. Epstein-Barr virus latent membrane protein-2A induces ITAM/Syk- and Akt-dependent epithelial migration through alphav-integrin membrane translocation. *J Virol* **2012**, *86*, 10308-10320, doi:10.1128/JVI.00853-12.
38. Fotheringham, J.A.; Mazzucca, S.; Raab-Traub, N. Epstein-Barr virus latent membrane protein-2A-induced DeltaNp63alpha expression is associated with impaired epithelial-cell differentiation. *Oncogene* **2010**, *29*, 4287-4296, doi:10.1038/onc.2010.175.
39. Lee, H.M.; Lo, K.W.; Wei, W.; Tsao, S.W.; Chung, G.T.Y.; Ibrahim, M.H.; Dawson, C.W.; Murray, P.G.; Paterson, I.C.; Yap, L.F. Oncogenic S1P signalling in EBV-associated nasopharyngeal carcinoma activates AKT and promotes cell migration through S1P receptor 3. *J Pathol* **2017**, *242*, 62-72, doi:10.1002/path.4879.
40. Fukuda, M.; Longnecker, R. Latent membrane protein 2A inhibits transforming growth factor-beta 1-induced apoptosis through the phosphatidylinositol 3-kinase/Akt pathway. *J Virol* **2004**, *78*, 1697-1705, doi:10.1128/jvi.78.4.1697-1705.2004.
41. Wang, L.; Tian, W.D.; Xu, X.; Nie, B.; Lu, J.; Liu, X.; Zhang, B.; Dong, Q.; Sunwoo, J.B.; Li, G.; et al. Epstein-Barr virus nuclear antigen 1 (EBNA1) protein induction of epithelial-mesenchymal transition in nasopharyngeal carcinoma cells. *Cancer* **2014**, *120*, 363-372, doi:10.1002/cncr.28418.
42. Saridakis, V.; Sheng, Y.; Sarkari, F.; Holowaty, M.N.; Shire, K.; Nguyen, T.; Zhang, R.G.; Liao, J.; Lee, W.; Edwards, A.M.; et al. Structure of the p53 binding domain of HAUSP/USP7 bound to Epstein-Barr nuclear antigen 1 implications for EBV-mediated immortalization. *Mol. Cell* **2005**, *18*, 25–36. https://doi.org/10.1016/j.molcel.2005.02.029.
43. Lu, J.; Murakami, M.; Verma, S.C.; Cai, Q.; Haldar, S.; Kaul, R.; Wasik, M.A.; Middeldorp, J.; Robertson, E.S. Epstein-Barr Virus nuclear antigen 1 (EBNA1) confers resistance to apoptosis in EBV-positive B-lymphoma cells through up-regulation of survivin. *Virology* **2011**, *410*, 64-75, doi:10.1016/j.virol.2010.10.029.
44. Fan, C.; Tang, Y.; Wang, J.; Xiong, F.; Guo, C.; Wang, Y.; Xiang, B.; Zhou, M.; Li, X.; Wu, X.; et al. The emerging role of Epstein-Barr virus encoded microRNAs in nasopharyngeal carcinoma. *J Cancer* **2018**, *9*, 2852-2864, doi:10.7150/jca.25460.
45. Cai, L.; Ye, Y.; Jiang, Q.; Chen, Y.; Lyu, X.; Li, J.; Wang, S.; Liu, T.; Cai, H.; Yao, K.; et al. Epstein-Barr virus-encoded microRNA BART1 induces tumour metastasis by regulating PTEN-dependent pathways in nasopharyngeal carcinoma. *Nat Commun* **2015**, *6*, 7353; Correction in *Nat Commun* **2020**, *11*, 3437.
46. Choy, E.Y.; Siu, K.L.; Kok, K.H.; Lung, R.W.; Tsang, C.M.; To, K.F.; Kwong, D.L.; Tsao, S.W.; Jin, D.Y. An Epstein-Barr virus-encoded microRNA targets PUMA to promote host cell survival. *J Exp Med* **2008**, *205*, 2551-2560, doi:10.1084/jem.20072581.
47. Wong, T.S.; Chen, S.; Zhang, M.J.; Chan, J.Y.; Gao, W. Epstein-Barr virus-encoded microRNA BART7 downregulates major histocompatibility complex class I chain-related peptide A and reduces the cytotoxicity of natural killer cells to nasopharyngeal carcinoma. *Oncol Lett* **2018**, *16*, 2887-2892, doi:10.3892/ol.2018.9041.
48. Song, Y.; Li, X.; Zeng, Z.; Li, Q.; Gong, Z.; Liao, Q.; Li, X.; Chen, P.; Xiang, B.; Zhang, W.; et al. Epstein-Barr virus encoded miR-BART11 promotes inflammation-induced carcinogenesis by targeting FOXP1. *Oncotarget* **2016**, *7*, 36783-36799, doi:10.18632/oncotarget.9170.
49. Huang, J.; Qin, Y.; Yang, C.; Wan, C.; Dai, X.; Sun, Y.; Meng, J.; Lu, Y.; Li, Y.; Zhang, Z.; et al. Downregulation of ABI2 expression by EBV-miR-BART13-3p induces epithelial-mesenchymal transition of nasopharyngeal carcinoma cells through upregulation of c-JUN/SLUG signaling. *Aging (Albany NY)* **2020**, *12*, 340-358, doi:10.18632/aging.102618.
